# Bullying Behaviors among Macanese Adolescents—Association with Psychosocial Variables

**DOI:** 10.3390/ijerph14080887

**Published:** 2017-08-07

**Authors:** Xue Weng, Wing Hong Chui, Liu Liu

**Affiliations:** 1 Department of Applied Social Sciences, City University of Hong Kong, Tat Chee Avenue, Kowloon, Hong Kong, China; eric.chui@cityu.edu.hk; 2 School of Social and behavioral Sciences, Nanjing University, Nanjing 210023, China; liuliu@nju.edu.cn

**Keywords:** bullying, victimization, adolescence, psychosocial variables, Macau

## Abstract

Bullying is a widespread public health problem among school students. Using a large sample of Macanese school adolescents, the present study examines psychosocial conditions and demographic characteristics in discriminating the following four subgroups of students: victims; bullies; bully-victims; and a comparison group of adolescents. Participants included 2288 adolescents from 13 primary and secondary schools in Macau whose ages ranged from 10 to 20 years. Statistical results revealed significant differences among the groups and indicated that adolescents who are involved in school bullying experience worse psychosocial adjustment. Specifically, among the four subgroups of students, bully-victims reported the strongest feelings of anxiety, depression, and negative affectivity, and expressed the lowest satisfaction with life. Compared with students who were not involved in bullying and victimization, bullies experienced more anxiety and depression and victims had lower levels of satisfaction with life. In addition, boys were more likely to engage in bullying behaviors and younger students had a greater probability of being victimized by their peers at school. Implications for future research and practice on bullying perpetration and the prevention of peer victimization are discussed.

## 1. Introduction

School bullying is recognized as a widespread phenomenon among Chinese societies. Studies on school bullying have been conducted in Mainland China [1,2,3], Hong Kong [4,5,6,7], and Taiwan [8,9,10]. According to a thorough review conducted by Chan and Wong [11], the prevalence of bullying victimization ranged from 2% to 66% in studies conducted in Mainland China, from 20% to 62% in studies conducted in Hong Kong, and from 24% to 50% in studies conducted in Taiwan, whereas the prevalence rate of bullying perpetration at school was found to range from 2% to 34% in Mainland China, from 19% to 56% in Hong Kong, and from 40% to 68% in Taiwan. Empirical research on school bullying in Macau is relatively rare. In a survey of 2232 Macanese school students, the prevalence rate of verbal bullying ranged from 26% to 78%, while the corresponding rate for physical bullying ranged from 16% to 46% [12].

Concerns about the prevalence of bullying are magnified by concerns about the adverse physical, social, and psychological effect on both the victims and perpetrators of bullying. There is evidence to show that bullying and victimization are strongly related to behavioral misconduct [13,14], poor physical health [15,16], psychosocial adjustment [17,18,19], mental health difficulties [20], and academic adjustment [21].

Recently, studies have moved beyond simply relating bullying to psychosocial factors. Instead, research on bullying has concentrated on distinguishing the group characteristics of those involved in bullying in terms of psychosocial symptoms. A four-category classification is widely used in studies of school bullying [18,22,23,24,25]. The four subgroups are (1) pure bullies, who bully others; (2) pure victims, who are the victims of others’ bullying; (3) bully-victims, who both bullies and are bullied by others; (4) comparisons, who are neither bullies nor victims. Negative behavioral and psychosocial consequences have been found across the first three bully/victim subgroups.

A substantial body of research has shown that bullying victimization is linked to anger [26,27], loneliness [28,29], social anxiety [13,30], depression [31,32,33], diminishing self-esteem [34], suicide ideation, and even attempted suicide [35,36]. Researchers have also found that peer victimization is related to reduced life satisfaction [37,38] and decreased quality of life [39]. The influence of peer victimization has adverse long-term consequences. Longitudinal research has shown that victims appear to suffer long-term psychosocial problems for years, even after the bullying has ended [40,41,42].

Bullying perpetration is also associated with heightened risk of psychosocial distress, including depressive symptoms [43], anxious feelings [44,45,46], anger [47], negative affectivity [48], suicidal ideation [36], and lower satisfaction with their lives [49,50]. Bullies appear to experience more psychiatric problems [51] and substance abuse problems [52]. Longitudinal studies have found that individuals who are bullies during childhood and adolescence are more likely to commit antisocial and/or criminal behaviors in adulthood [53,54,55].

Bully-victims have been identified as an extremely problematic group. Compared to those who are only aggressive or who are only victimized, they experience more psychosocial symptoms [23]. In particular, previous research has found that bully-victims exhibit higher rates of problem behavior [24,25,56,57] and academic failure [58], and express more psychosocial distress, such as depression and anxiety [23,33,56,59], negative moods [60], and psychiatric symptoms [51]. Nevertheless, few studies have investigated life satisfaction among bully-victims.

Although bullying behavior is widespread among Macanese students, Macanese research on school bullying is scarce. To the best of our knowledge, no studies have investigated the demographic and psychosocial differences between victims, bullies, bully-victims, and comparisons using a Macanese sample. To address the existing gaps, this study uses a multivariate research design to investigate the association between bullying perpetration, peer victimization, and psychosocial variables among a large sample of Macanese adolescents. It is hypothesized that demographic and psychosocial dimensions differentiate between subgroups of adolescents exhibiting pure versus combined forms of bullying and victimization (i.e., bullies, victims, bully-victims, and comparisons).

## 2. Method

### 2.1. Sample and Procedure

Macau was a colony of the Portuguese government for more than 400 years (1557–1999) and is now a special administrative region (SAR) of the People’s Republic of China. It is a small territory, and 92.4% of its population is of Chinese descent. As Macau was subject to Portuguese sovereignty for more than a century, Western influences in the territory are substantial. However, Macanese people still maintain traditional Chinese values and lifestyles. Macau is therefore a melting pot of Chinese and Western cultures.

In this study, students attending primary school (Primary 6; equivalent to Grade 6 in the US), middle school (forms 1–3, equivalent to grades 7–9 in the US), and high school (forms 4–6, equivalent to grades 10–12 in the US) were targeted. The chosen schools and classes were randomly sampled according to the existing list of all schools issued by the Macau Education Bureau. It is hoped that the final sample of this study covered both early and late adolescents. Upon permission being granted by the university’s research ethics committee and the councils of the schools involved, the parents or guardians of the adolescents selected to take part in the survey were sent a consent form informing them of the purpose of the project and that their child had been selected for inclusion in the study. The investigation was conducted between April 2015 and November 2015. All data were collected through an anonymous, self-administered questionnaire in a classroom setting during school hours; the process was supervised by trained research assistants.

A total of 2407 adolescents from 13 primary and secondary schools in Macau participated in the survey. Respondents were excluded from the present analysis if they gave too many incomplete or incoherent responses (*n* = 101) or if their ethnicity was not Chinese (*n* = 18); this process yielded a final analytic sample of 2288. The mean age of the sample was 15.47 years (SD = 2.05). The sample comprised 906 male students (39.6%) and 1382 female students (60.4%): 69 were Primary 6 students (3.0%), 275 were Form 1 students (12.0%), 338 were Form 2 students (14.8%), 398 were Form 3 students (17.4%), 459 were Form 4 students (20.1%), 474 were Form 5 students (20.7%), and 266 were Form 6 students (11.6%).

### 2.2. Measures

#### 2.2.1. Bullying/Victimization

The items assessing bullying and victimization were measured with the Chinese version [61] of the Illinois Bully Scale (IBS) [47]. The nine-item bully scale assessed the participants’ self-reported bullying behaviors in the previous 30 days, including teasing, social exclusion, name-calling, and rumor spreading. Similarly, the experience of being victimized was assessed by a four-item victimization scale from the IBS (i.e., “other students called me names”, “other students made fun of me”, “other students picked on me”, and “I got hit and pushed by other students”). Response options included “never”, “one or two times”, “three or four times”, “five or six times”, and “seven or more times”. A higher score on the bullying scale and the victimization scale indicates a greater tendency to engage in bullying behaviors and to be bullied at school, respectively. The internal consistency of the IBS was high (bullying: Cronbach’s *α* = 0.84; victimization: Cronbach’s *α* = 0.72) in the present study.

#### 2.2.2. Psychosocial Variables

Life satisfaction was assessed with the Satisfaction with Life Scale [62]. This scale consists of five items (e.g., “In most ways, my life is close to my ideal”) rated on a seven-point Likert scale ranging from “1 = strongly disagree” to “7 = strongly agree”. The scale is one of the most commonly used scales for measuring general life satisfaction. Higher scores indicate greater life satisfaction. In the present study, the scale had a high internal consistency, with a Cronbach’s alpha of 0.88.

The anxiety/depression scale was drawn from the Chinese version of the 12-item General Health Questionnaire (GHQ-12) [63,64]. This scale consists of four self-report items, and in the current study, the students were asked to report symptoms of depression and anxiety they had experienced during the past week. Response options range from 0 (less than usual) to 3 (much more than usual). Higher scores signify greater levels of anxiety and depression. In this study, the scale showed high internal consistency (Cronbach’s α = 0.84).

The Positive and Negative Affect Schedule (PANAS) [65] consists of 20 adjectives, with 10 items reflecting positive affect and 10 items describing negative affect. Positive affectivity (PA) represents an individual’s pleasurable emotions; individuals with PA are typically interested, excited, enthusiastic, and active. Negative affectivity (NA) comprises a broad range of aversive mood states, such as distress, upset, nervousness, guilt, and fear. The respondents were asked to indicate to what extent they had felt positive and negative feelings within the last two weeks on a five-point Likert scale (1 = very slightly/not at all, 2 = a little, 3 = moderately, 4 = quite a bit, 5 = extremely). Sufficient internal consistency was found for both the PA scale (Cronbach’s α = 0.83) and the NA scale (Cronbach’s α = 0.89).

#### 2.2.3. Demographic Information

Several sociodemographic variables were included to protect against concerns about spuriousness. The demographic covariates were age, gender, grade level (Primary 6 to Form 6), and school academic performance (poor, average, good).

Means, standard deviations, and ranges for the study variables are presented in Table 1.

### 2.3. Statistical Analyses

Statistical analyses were conducted using SPSS version 22.0 (SPSS, Inc., Chicago, IL, USA). Bivariate analyses were first conducted to determine whether the students’ bullying categories (i.e., bully, victim, bully-victim, and comparison) were associated with their age, gender, grade, academic performance (i.e., poor, average, good), and psychosocial variables (i.e., life satisfaction, anxiety/depression, positive and negative affect). One-way ANOVAs and a Scheffé’s post hoc test were used for the continuous variables, and a Chi-square test was used for the categorical variables. Then, a multinomial logistic regression was conducted to assess whether the abovementioned factors differentiated the distributions of the bully, victim, bully-victim, and comparison subgroups. The comparison adolescents, who were neither bullies nor victims, were the reference group in this analysis.

## 3. Results

### 3.1. Bivariate Analyses

Students were classified as comparisons, victims, bullies, or bully-victims on the basis of their self-reports of bullying and victimization. In line with previous research [23], the study used the cutoff point of “three or four times” to code a student as involved or not involved in each form in bullying or victimization. That is, students were classified as bullies, victims, or bully/victims only if they were involved in at least one behavior indicative of bullying and/or victimization three or four times or more often. Bullies (*n* = 372, 16.3%) reported bullying others three or more times and never or rarely being victimized. Victims (*n* = 406, 17.7%) reported having been victimized three or more times and never or rarely bullying others. Bully-victims (*n* = 775, 33.9%) reported having been both bullied and victimized three or more times. Those classified as comparisons (*n* = 675, 29.5%) reported never or rarely bullying or being victimized. According to the four-group classification, the bully-victim subgroup was the most prevalent, while the pure bully subgroup was the least prevalent.

Table 2 shows the percentages of students in each of the bully/victim categories by gender, grade, and academic performance. Chi-square analyses indicated that there were significant gender differences across groups (*χ*^2^ = 28.54; *p* < 0.001). Specifically, boys were more represented in the bully-victim group (40.8% of all male students) than girls (30.9% of all female students). Further, girls were more represented in the victim group (19.6% of all female students) and the comparison group (33.3% of all female students) than boys (victims: 16.2%, comparisons: 19.6%). The percentages of males and females in the bully group did not differ significantly (17.3% of all male students; 16.3% of all female students). 

Chi-square analyses yielded significant differences for grade levels across the bully/victim categories (*χ*^2^ = 40.15; *p* < 0.01). In every grade except for Form 4, the bully-victim group had the highest percentage of pupils compared to the other three groups. Primary 6 students had the lowest percentage in the bully group (9.0%), followed by Form 1 (12.9%), compared to a range of 15.7% to 19.7% among students in the other grades. With respect to the victim group, approximately 32.8% of the Primary 6 students and 27.3% of the Form 1 students were classified in this category, in contrast to a range of 15.9% to 17.9% among the students in the other grades.

Chi-square analyses also signified that there were significant group differences by school academic performance (*χ*^2^ = 18.70; *p* < 0.005). Specifically, a significantly greater prevalence of poor academic performance was reported in the bully-victim group (38.3%) than in the other three groups. There was a significantly lower prevalence of average academic performance in the bully (15.1%) and bully-victim (32.1%) groups.

Given that the bully/victim groups differed across gender, grade, and academic performance, these demographic variables were considered as control variables in subsequent analyses.

Next, we used one-way ANOVAs to examine whether the mean value of age and psychosocial variables (e.g., life satisfaction, anxiety/depression, PA, and NA) differed by bully/victim subgroup. Post hoc tests showed that age and psychosocial variables differed significantly across bully/victim groups (see Table 3). In particular, victims were significantly younger than bullies, bully-victims, and comparisons. There were no significant differences in age among the other three groups. Students who were in the comparison subgroup reported significantly higher levels of life satisfaction (M = 22.97) compared to those in the victim and bully-victim subgroups (M = 21.70 and 20.97, respectively). Students in the bully-victim category experienced significantly higher levels of anxiety/depression (M = 9.02) than those in the pure victim and comparison groups (M = 21.70 and 8.02, respectively). Bully-victims reported the highest levels of NA (M = 28.15) among the groups, and, interestingly, they also reported higher levels of PA (M = 30.12) than adolescents in the pure victim and comparison groups (M = 28.61 and 28.57, respectively). In addition, bullies also reported higher PA (M = 29.87) and more anxiety/depression (M = 8.66) than the comparison group.

### 3.2. Multivariate Analyses

Following the bivariate analyses, relationships among demographic and psychosocial indicators and bully/victim status using the multinomial logistic regression are presented in Table 4. To aid interpretation, Table 4 also shows standardized coefficients for the logistic regression [66]. (The formula is *β* = b(*s_x_*)(R)/*s*_logit(*Ŷ*)_, where b is the unstandardized logistic regression coefficient, R the correlation between the observed and predicted values of the dependent variable Y, *s_x_* is the standard deviation of *x*, and *s*_logit(Ŷ)_ is the estimated standard deviation of the outcome variable. Detailed information on the calculation of fully standardized coefficients is available in Menard [66]). The standardized coefficients allow for an assessment of the relative effect magnitude of the explanatory variables in a particular model. In this analysis, we used the comparison group, the members of which were not bullied and did not bully others, as the reference group. Age, gender, and psychosocial variables were all associated with involvement in bullying, but in different ways for bullies, victims, and bully-victims. There was no significant difference for grade and academic performance in any of the bully/victim categories.

Compared to the younger adolescents, the older adolescents were less likely to be bullies (OR = 0.85) or victims (OR = 0.88). Compared to the girls, the boys were significantly more likely to be bullies (OR = 1.57) or bully-victims (OR = 1.83).

The bullies, victims, and bully-victims demonstrated a poorer psychosocial condition than the comparison youths. Specifically, victims and bully-victims reported significantly worse life satisfaction (OR = 0.96 and 0.96, respectively). Bullies, victims, and bully-victims also experienced more anxiety and depression, though this relationship was not statistically significant. Relative to the comparison group, being a bully or a bully-victim was associated with increased odds of NA (OR = 1.03 and 1.05, respectively). One interesting finding is that PA was also positively related to being a bully (OR = 1.04) or a bully-victim (OR = 1.06).

## 4. Discussion

Bullying is a prevalent public health problem among school students. This study divided the sample into four subgroups to investigate demographic and psychosocial differences between victims, bullies, bully-victims, and comparison adolescents. The novel findings of this study contribute to our understanding of the bullying phenomenon in a rarely researched school population, namely Macanese adolescents. The majority of the students (33.9%) in our study were recognized as being both victims and perpetrators of bullying. Roughly equal proportions of these students had been frequently victimized by bullies (17.7%) or had frequently bullied others (16.3%); the remaining 29.5% of the students were neither perpetrators nor victims. The prevalence of victims was higher and the prevalence of bullies was lower than in Chan and Wong’s [5] survey among 1880 Hong Kong students (bullies: 51.8%; victims: 2.5%), whereas the prevalence of bully-victims was similar (32.1%).

The study first examined whether demographic characteristics differentiate across the bully/victim categories. Gender differences were found. Consistent with previous studies [5,33,46,58,67,68,69,70,71], being male was associated with increased odds of being a bully or a bully-victim. Similar to the report by Pouwelse, Bolman, Lodewijkx, and Spaa [72], but in contrast to the study of Griffin and Gross [73], significantly more pure victims were observed among female adolescents. The overrepresentation of girls in the comparison group is in line with prior research [69,72].

In terms of age difference, our findings indicate that junior students (Primary 6 and Form 1) have a higher rate of victimization but a lower rate of bullying than senior high students. We also found that students who were pure victims were significantly younger than the students in the other three subgroups. Multivariate findings indicated that the older the student is, the less likely he or she is to be a perpetrator or a victim. This is congruent with Chinese [1,2,4] and Western [74,75,76] studies that have found that younger students have a greater probability of being victimized by their peers at school. One possible reason for this is that senior students undergo more physical and psychosocial developmental than their younger counterparts, and so they are more able to protect themselves from peer victimization [77]. The study found that bully-victims are more likely to be associated with poor academic performance, which also lends support to prior research [18,58].

With respect to psychosocial differences, our results confirmed the findings of earlier studies that adolescents who are involved in bullying have higher rates of psychosocial problems [44,45,46,72,78,79,80,81]. Specifically, we found that pure bullies and bully-victims reported more anxious and depressive feelings than comparison adolescents [44,45,46,72,80]. Consistent with previous studies [37,38], we found that those who experienced bullying victimization (i.e., pure victims, bully-victims) manifested lower levels of life satisfaction.

In the present study, we also examined two opposite mood states, negative affectivity (NA) and positive affectivity (PA). These two variables have seldom been explored in the previous literature on school bullying. In a study by Karatzias et al. [48], victims reported higher NA than bullies and noninvolved peers, whereas PA failed to differentiate the groups of bullies, victims, and bully-victims. Nevertheless, we found that adolescents who engaged in bullying (i.e., bullies, bully-victims) reported a significantly higher level of PA and NA than pure victims and comparisons. Watson and Clark’s [82] review of studies on NA indicated that individuals with high NA are more likely to be perceived as hostile, demanding, and distant, as they often experience negative mood states. Since bullying is a subtype of aggressive behavior, research has found that high-NA individuals are more likely to be involved in aggressive activities in some situations [83]. Meanwhile, it has also been found that bullies have superior psychosocial well-being and enjoy high social standing among students [46]. Developmental studies have indicated that high social standing is strongly related to positive self-image and psychological well-being [84]. Furthermore, Chinese students who are influenced by Confucian and collectivistic tradition often perceive bullying as a collective conduct [77]. Through the expression of aggression, bullies not only can obtain a powerful and dominant position in their peer group [48,85], but also to maintain group conformity [77,86]. This may be one of the possible reasons why, in the current study, bullies and bully-victims reported higher PA and NA scores at the same time. Negative mood status triggers aggressive behavior, such as bullying others; in return, the expression of bullying behavior helps them obtain high social status, group interests, and a superior self-view. Future studies should focus on the relationships between PA, NA, and bullying experience. Future research should also consider the cultural contexts of school bullying in Chinese societies.

This study also had limitations. First and foremost, the current study exclusively depended on the self-reports of students, which may undermine the validity of the results [87]. Students are less likely to be honest in regard to direct questions about recent bullying and victimization experience. Students may deny this negative experience, which may result in social desirability biases. Social desirability bias refers to the notion that survey respondents tend to under-report behaviors deemed undesirable by society, while they tend to over-report behaviors viewed as socially desirable [88]. Using a multi-method approach is recommended for future research. For example, the peer nomination method directly asks classmates about the roles students play. Peer report is an alternative approach to identifying victims and bullies on the basis of a consensus of a large group of students, and so it provides a more reliable estimate of peer social status [89]. Second, the cross-sectional nature of the study meant that it was unable to examine the causal relationships between school bullying and psychosocial outcomes. On the one hand, psychosocial maladjustment could have occurred prior to school bullying, and perhaps even could have triggered school bullying. On the other, bullying and victimization could result in poor psychosocial symptoms. It is likely that reciprocal relations of causality exist and that the path is not in the same direction. Thus, in future investigations, longitudinal studies are necessary to examine the lagged effects of bullying and victimization on psychosocial problems.

In terms of recommendations for school practice, prevention and intervention efforts should not only identify pure bullies and pure victims, but should also focus more on students who adopt the roles of both bully and victim as such individuals display worse psychosocial well-being. Sociodemographic characteristics should be taken into account in school practice. Considering that younger students are more vulnerable to bullying victimization, it is essential that schools deliver an anti-bullying program to children at an early age. Anti-bullying measures are also supposed to be gender specific. Since psychosocial variables are strongly correlated with bullying and victimization, teachers, school counselors, and social workers should teach students emotional regulation and coping strategies to handle negative emotions and life events.

## 5. Conclusions

This is one of the first studies to examine demographic and psychological differences across the bully/victim continuum among Macanese primary and secondary students. Students who were involved in bullying and victimization reported poorer psychosocial conditions than other three sub-groups of students in this study. Our results show that bully-victims were the most vulnerable group that reported to experience a higher level of anxiety, depression, and negative affectivity and life satisfaction. Prevention and intervention strategies should better understand the differences between the bully/victim subgroups so as to address the specific mental health needs of bullies, victims and bully-victims. Helping professions such as teachers, school counsellors and social workers should equip students with useful stress-coping techniques and skills to minimize the negative psychosocial consequences of bullying.

## Figures and Tables

**Table 1:** Descriptive statistics for sample (*n* = 2288).

Variable	*n* (%) ^a^	Mean (SD)	Range
Age (in years)		15.47 (2.05)	10–20
Gender			
Male	906 (39.6)		
Female	1382 (60.4)		
Grade level			
Primary 6	69 (3.0)	11.16 (.59)	
Form 1	275 (12.0)	12.77 (1.02)	
Form 2	338 (14.8)	14.23 (1.24)	
Form 3	398 (17.4)	15.10 (1.16)	
Form 4	459 (20.1)	16.01 (1.17)	
Form 5	474 (20.7)	16.98 (1.15)	
Form 6	266 (11.6)	17.91 (1.04)	
Academic performance			
Poor	749 (32.7)		
Average	1072 (46.9)		
Good	459 (20.1)		
Life Satisfaction		21.86 (6.33)	5–35
Anxiety/Depression		8.55 (2.95)	4–16
Positive affect		29.31 (6.90)	10–50
Negative affect		26.03 (8.38)	10–50

Note: ^a^ Total percentages within this column may not be exactly 100% due to missing data.

**Table 2:** Demographic variables across bully/victim groups.

	Bully Status				*χ*^2^	*p*
	Bully	Victim	Bully-Victim	Comparison		
Total	372 (16.7%)	406 (18.2%)	775 (34.8%)	675 (30.3%)		
By gender					28.54	<0.001
Male	152 (17.3%)	142 (16.2%)	358 (40.8%)	226 (25.7%)		
Female	220 (16.3%)	264 (19.6%)	417 (30.9%)	449 (33.3%)		
By grade					40.15	<0.005
Primary 6	6 (9.0%)	22 (32.8%)	23 (34.3%)	16 (23.9%)		
Form 1	34 (12.9%)	72 (27.3%)	91 (34.5%)	67 (25.4%)		
Form 2	56 (17.0%)	59 (17.9%)	124 (37.6%)	91 (27.6%)		
Form 3	61 (15.7%)	63 (16.2%)	144 (37.1%)	120 (30.9%)		
Form 4	77 (17.2%)	71 (15.9%)	146 (32.7%)	153 (34.2%)		
Form 5	91 (19.7%)	75 (16.3%)	152 (33.0%)	143 (31.0%)		
Form 6	47 (17.9%)	42 (16.0%)	91 (34.6%)	83 (31.6%)		
By academic performance					18.70	<0.005
Poor	134 (18.5%)	130 (17.9%)	278 (38.3%)	184 (25.3%)		
Average	157 (15.1%)	199 (19.1%)	335 (32.1%)	351 (33.7%)		
Good	81 (17.9%)	77 (17.0%)	157 (34.7%)	137 (30.3%)		

**Table 3:** Comparison of independent variables across bully/victim groups.

	Bully Status			
	Bully	Victim	Bully-Victim	Comparison
Age	15.59 _a_	15.08	15.48 _a_	15.65 _a_
Life Satisfaction	21.97 _a,b_	21.70 _a_	20.97 _a_	22.97 _b_
Anxiety/Depression	8.66 _a,b_	8.39 _a,c_	9.02 _b_	8.02 _c_
Positive affect	29.87 _a,b_	28.61 _a,c_	30.12 _b_	28.57 _c_
Negative affect	26.70 _a_	25.06 _a,b_	28.15	23.82 _b_

Notes: Means in the same row that do not share subscripts significantly differ from each other (Scheffé test, *p* < 0.05).

**Table 4:** Results of the multinomial logistic regression analysis.

Variable	Bullies		Victims		Bully-Victims	
	*β*	OR (95% CI)	*β*	OR (95% CI)	*β*	OR (95% CI)
Age	**−0.34**	**0.84 (0.74, 0.96)**	**−0.25**	**0.88 (0.78, 1.00)**	−0.12	0.94 (0.85, 1.05)
Gender						
Male	**0.21**	**1.55 (1.16, 2.08)**	0.06	1.13 (0.84, 1.51)	**0.28**	**1.79 (1.40, 2.29)**
Female (ref)						
Grade						
Primary 6	−0.20	0.30 (0.08, 1.14)	0.06	1.44 (0.46, 4.44)	0.05	1.37 (0.49, 3.83)
Form 1	−0.20	0.47 (0.19, 1.13)	0.06	1.20 (0.52, 2.77)	0.05	1.16 (0.56, 2.39)
Form 2	−0.17	0.61 (0.30, 1.24)	−0.10	0.76 (0.37, 1.52)	0.03	1.08 (0.60, 1.96)
Form 3	−0.21	0.57 (0.31, 1.06)	−0.14	0.69 (0.38, 1.28)	−0.02	0.95 (0.57, 1.58)
Form 4	−0.15	0.69 (0.40, 1.17)	−0.12	0.74 (0.43, 1.26)	−0.06	0.86 (0.55, 1.35)
Form 5	−0.04	0.91 (0.56, 1.47)	−0.07	0.85 (0.52, 1.39)	−0.03	0.93 (0.62, 1.40)
Form 6 (ref)						
Academic performance						
Poor	0.10	1.24 (0.84, 1.84)	0.11	1.28 (0.86, 1.92)	0.08	1.19 (0.85, 1.67)
Average	−0.06	0.89 (0.63, 1.27)	0.05	1.10 (0.77, 1.56)	−0.02	0.96 (0.71, 1.29)
Good (ref)						
Life Satisfaction	−0.14	0.98 (0.95, 1.00)	**−0.25**	**0.96 (0.94, 0.98)**	**−0.25**	**0.96 (0.94, 0.98)**
Anxiety/Depression	0.10	1.03 (0.97, 1.10)	0.01	1.00 (0.94, 1.07)	0.14	1.05 (0.99, 1.11)
Positive affect	0.26	1.04 (1.02, 1.07)	0.08	1.01 (0.99, 1.04)	0.36	1.06 (1.04, 1.08)
Negative affect	0.22	1.03 (1.00, 1.05)	0.10	1.01 (0.99, 1.03)	0.39	1.05 (1.03, 1.07)

Note: *β* = standardized logistic regression coefficients. The reference category in this analysis was the comparison group, whose members were not involved in bullying or victimization. Significant findings, indicated by 95% confidence intervals that do not include 1, are presented in bold.
